# The Roles of Albumin Levels in Head and Neck Cancer Patients with Liver Cirrhosis Undergoing Tumor Ablation and Microsurgical Free Tissue Transfer

**DOI:** 10.1371/journal.pone.0052678

**Published:** 2012-12-28

**Authors:** Huang-Kai Kao, Wei F. Chen, Chih-Hao Chen, Victor Bong-Hang Shyu, Ming-Huei Cheng, Kai-Ping Chang

**Affiliations:** 1 Department of Plastic and Reconstructive Surgery, Chang Gung Memorial Hospital, Chang Gung University College of Medicine, Tao-Yuan, Taiwan; 2 Division of Plastic and Reconstructive Surgery, Department of Surgery, University of Iowa Hospitals and Clinics, Iowa City, Iowa, United States of America; 3 Department of Otolaryngology-Head and Neck Surgery, Chang Gung Memorial Hospital, Chang Gung University College of Medicine, Tao-Yuan, Taiwan; Harvard Medical School, United States of America

## Abstract

**Objective:**

To evaluate the changes of serum albumin levels during the peri-operative period, and correlate these changes to surgical outcomes, postoperative morbidity and mortality in head and neck cancer patients with cirrhosis.

**Methods:**

57 patients with liver cirrhosis out of 3,022 patients who underwent immediate free flap reconstruction after surgical ablation of head and neck cancer performed over a 9-year period were included in the study. Two sets of groups were arranged based on the preoperative albumin (>3.5 g/dL vs. ≤ 3.5 g/dL) and POD1 albumin (>2.7 g/dL vs. ≤ 2.7 g/dL) levels and were compared with respect to patient-related variables, surgical outcomes, medical and surgical complications, and mortalities.

**Results:**

All patients had significant decreases in albumin levels postoperatively. Hypoalbuminemia, both preoperative and postoperative, was associated with the model for end-stage liver disease (MELD) score, the amount of blood loss, the duration of ICU stay and hospital stay, and postoperative medical and surgical complications. In particular, preoperative hypoalbuminemia (serum albumin ≤ 3.5 g/dL) was associated strongly with medical complications and mortality, while postoperative hypoalbuminemia (serum albumin ≤ 2.7 g/dL) with surgical complications.

**Conclusion:**

Our study demonstrated the prognostic values of albumin levels in head and neck cancer patient with liver cirrhosis. The perioperative albumin levels can be utilized for risk stratification to potentially improve surgical and postoperative management of these challenging patients.

## Introduction

Medical conditions such as malnutrition, cancer, advanced age, and other preexisting diseases all contribute to hypoalbuminemia. Due to the location involving the upper digestive tract, the patients with head and neck cancer are among the most malnourished of cancer patients, with the reported prevalence up to 35–50% [1], [2]. Besides, malnutrition and hypoalbuminemia are also common findings in patients with liver cirrhosis. Thus, in patients with head and neck cancer and concomitant liver cirrhosis, hypoalbuminemia is even more aggravated and presents a challenging issue that warrants further workup and management.

In our previous study, the incidence of liver cirrhosis among head and neck cancer patients in Taiwan was 2% during a 9-year follow-up period [3]. From a theoretical perspective, head and neck cancer patients with liver cirrhosis may be at an even greater risk of malnutrition that adversely affect surgical outcomes because of greater disease severity and comorbidity than the individual disease only.

There are many tools to assess patients’ nutritional status. Serum albumin is a good and simple predictor of surgical risk and has a close correlation with the degree of malnutrition [4]. However, there is no evidence regarding the effect of albumin variability on the development of postoperative complications in cirrhotic patients undergoing free-flap surgery for head and neck cancer. This study tested the hypothesis that peri-operative serum albumin concentration, a simple nutritional marker of protein metabolism and endogeneous liver function, correlates with postoperative surgical outcome, medical morbidity, and mortality, using exclusively a group of head and neck cancer patients with cirrhosis receiving tumor ablative and free-flap surgery.

## Methods

### Patients

3,022 microsurgical free flap reconstructions after head and neck cancer ablation were performed at Chang Gung Memorial Hospital - Linkou Medical Center, Taiwan, between January 2001 and December 2009. By the standard protocol of our institute, all patients underwent head and neck cancer ablation following free tissue transfer receiving routinely abdominal ultrasonography, bone scan, magnetic resonance image, chest x ray, blood count, serum chemistry tests, and coagulation profiles preoperatively. The patients with head and neck cancer who underwent the operation procedure included both tumor resection with/without neck dissection and microsurgical free tissue transfer and had positive findings of liver cirrhosis in the preoperative survey by abdominal ultrasonography were included in the study. On the other hand, 42 (1.4%) of 3,022 patients were excluded from study because the abdominal ultrasonography were not performed preoperatively. Fifty-seven patients were diagnosed with cirrhosis and were enrolled in the study. These included 55 males (96.8%) and 2 females (3.2%). This study was approved by the institutional review board of Chang Gung Memorial Hospital – Linkou Medical Center. Informed consent was not needed since the data were analyzed anonymously and retrospectively. The institutional review board specifically waived the need for consent.

### Patients’ Data

Serum albumin concentrations were measured at 3 time points - preoperatively, on postoperative day 1 (POD1), and on postoperative day 7 (POD7). We considered the range of 3.5–5.0 g/dL as normal according to our laboratory reference. Patient-related variables included age, gender, body mass index (BMI), tumor stage, tumor location, and pre-existing disease. Operative variables included operative time, intraoperative blood loss, blood transfusion rate, duration of intensive care unit stay, and duration of hospital stay. To assess the severity of liver cirrhosis, the model for end-stage liver disease (MELD) score was employed based on laboratory values preoperatively and defined as the sum of following formula: 3.78[Ln serum bilirubin (mg/dL)]+11.2[Ln INR] +9.57[Ln serum creatinine(mg/dL)] +6.43 [5].

### Definition of Morbidity and Mortality

Positive events for postoperative morbidity were defined as the patient having at least one of the major medical or surgical complications listed below. Medical morbidity was defined as any complication that resulted in the following organ dysfunctions, including cardiovascular (acute myocardial infarct), pulmonary (pneumonia, pleural effusion, acute respiratory distress syndrome), gastrointestinal (esophageal varices bleeding), renal (acute renal failure) complications, hepatic encephalopathy and sepsis [6]. Surgical morbidities were recipient-site infection and flap-related complications. Flap-related complications included partial or complete flap loss and re-exploration during the first postoperative week due to arterial or venous circulatory compromise [3]. The morbidity and mortality events analyzed were restricted to the postoperative period during the operation-specific hospital stay.

### Statistical Analysis

Data manipulation and statistical analyses were conducted using SAS software version 9.1 (SAS Institute Inc., Cary, NC, USA). All statistics are presented as mean ± standard deviation for continuous variables unless otherwise specified. Differences in characteristics between groups were tested using the χ^2^ test for dichotomous variables and Wilcoxon match test or Wilcoxon rank sum test for continuous variables, as appropriate. The significance of differences between preoperative and POD1 albumin concentration was evaluated using the paired *t* test. Relations between the percentages of serum albumin level drops and other variables were assessed using linear regression analysis and the Pearson correlation coefficient. Logistic regression model was used to define the risk factors for postoperative morbidity and mortality. All clinical factors were calculated in a univariate analysis for crude odds ratio and put into the multivariate analysis to estimate the adjusted odds ratio for prediction of postoperative morbidity and mortality. All *p* values were 2-sided and the significance level was set at *p*<0.05.

## Results

### Comparisons of Patients Based on Preoperative and POD1 Serum Albumin Concentrations

The preoperative albumin levels ranged from 1.9 to 5.2 g/dL with a median of 3.9 (3.80±0.66) and POD1 albumin levels ranged from 1.6 to 3.5 g/dL with a median of 2.7 (2.63±0.45). Patients were divided into 2 preoperative groups based on preoperative albumin concentrations - those with low albumin levels (≤ 3.5 g/dL) and those with normal levels (>3.5 g/dL). In addition, 2 postoperative groups were stratified based on POD 1 median albumin value of 2.7 g/dL - those with levels ≤ 2.7 and those with levels >2.7 g/dL. Comparisons of patient demographics and operative variables based on preoperative albumin (>3.5 g/dL vs. ≤ 3.5 g/dL) and POD1 albumin (>2.7 g/dL vs. ≤ 2.7 g/dL) concentrations are presented in Table 1 and 2, respectively. Patients with preoperative albumin levels ≤ 3.5 g/dL had a significantly lower BMI and required longer ICU and hospital stays than those with normal albumin levels. No differences were seen in operative time, amount of blood loss, and the need for blood transfusion (Table 1). Similar to the preoperative comparison, patients with POD1 albumin concentrations ≤ 2.7 g/dL had a significantly lower BMI, and required longer ICU and hospital stays. The same group experienced a significantly higher rate of blood transfusion and more blood loss during operation when compared to patients with POD1 albumin >2.7 g/dL (Table 2).

**Table 1:** Patients’ demographics and operative variables based on preoperative albumin level.

	Albumin >3.5 (g/dL)	Albumin ≤3.5 (g/dL)	*p* value
	(*n* = 38)	*(n* = 19)	
**Age,** years	52.1±9.9	57.0±8.2	0.07
**Male/Female**	37/1	18/1	
**BMI** a	23.39±1.28	21.85±1.28	<0.001*
**Stage**			
II	14	9	0.57
III	6	2	0.71
IV	18	8	0.78
**Site**			
Buccal	13	5	0.76
Gum	8	3	0.73
Palate	0	3	0.03*
Tongue	8	3	0.73
Hypopharynx	3	3	0.39
Larynx	1	0	1
Mouth floor	3	1	1
Lip	2	1	1
**Operation variables**			
Operative time (range), min	734.6±179.4 (425–1150)	753.6±180 (426–1050)	0.71
Blood loss (range), mL	491.8±393.5 (100–1700)	515.8±293.5 (200–1250)	0.82
ICU^b^ Stay (days)	10.3±9.4 (5–47)	19.1±16.4 (5–62)	0.01*
Hospital Stay (days)	32.0±13.7 (3–65)	42.0±15.8 (16–75)	0.02*

a BMI, body mass index;

b ICU, intensive care unit;

* *P*<0.05.

**Table 2:** Patients demographics and operative variables based on POD1 albumin level.

	Albumin >2.7 (g/dL)	Albumin ≤ 2.7 (g/dL)	*p* value
	(n = 30)	(n = 27)	
**Age,** years	53.9±10.1	53.6±9.2	0.93
**Male/Female**	28 (93.3)	27 (100)	0.49
**BMI** a	23.3±1.44	22.4±1.3	0.02*
**Stage**			
II	10	12	0.43
III	3	5	0.46
IV	16	11	0.43
**Site**			
Buccal	9	8	1.00
Gum	8	3	0.19
Palate	0	3	0.10
Tongue	4	6	0.49
Hypopharynx	2	3	0.66
Larynx	1	1	1.00
Mouth floor	4	2	0.67
Lip	1	2	0.60
**Operation variables**			
Operative time, median (range), min	723.3±196.0	760.6±157.5	0.43
Blood loss (range), mL	383.0±288.7	629.6±392.5	0.01*
ICU^b^ Stay (days)	7.2±2.0	18.7±15.6	<0.001*
Hospital Stay (days)	28.3±13.4	42.7±13.7	<0.001*

a BMI, body mass index;

b ICU, intensive care unit;

* *P*<0.05.

### Correlation of Preoperative Albumin Concentration and Severity of Cirrhosis

**Figure 1:**
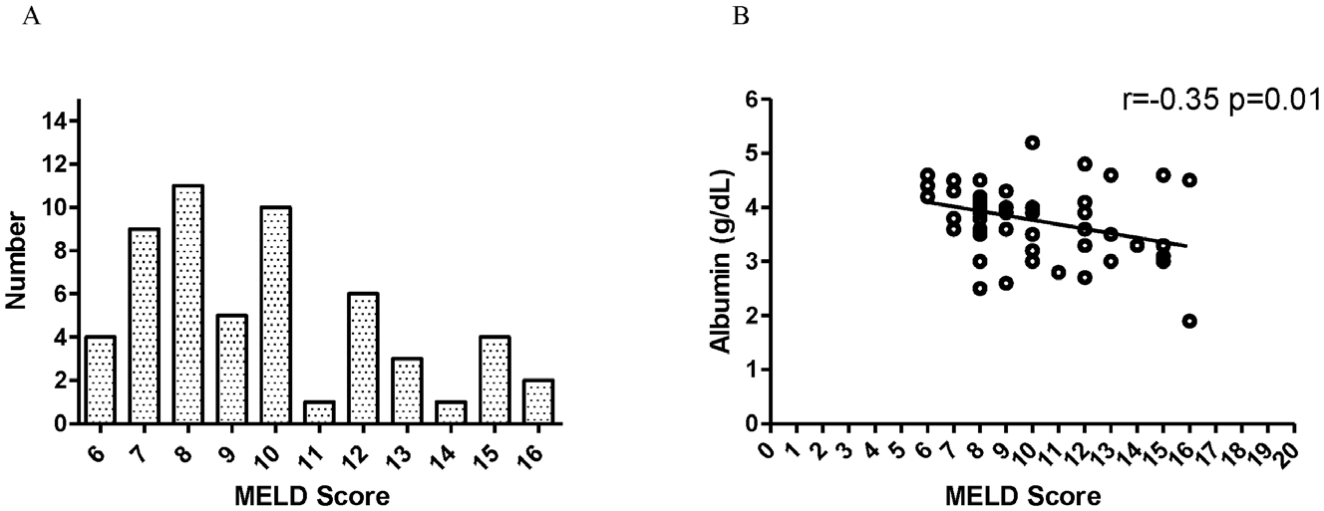
Correlation of preoperative albumin concentration and severity of cirrhosis. (A) The distribution of MELD scores in all patients. (B) MELD scores were negatively correlate with preoperative albumin levels (*r* = −0.35, *p* = 0.01). Patients with lower albumin concentrations tended to have higher MELD scores.

The severity of cirrhosis was evaluated using the MELD score. The distribution of MELD score among patients is demonstrated in Fig. 1A. MELD scores were found to negatively correlate with preoperative albumin levels (*r* = −0.35, *p* = 0.01). Patients with lower albumin concentrations tended to have higher MELD scores, suggesting more advanced cirrhotic states (Fig. 1B).

### Serial Change of Albumin Levels in the First Week Postoperatively

**Figure 2:**
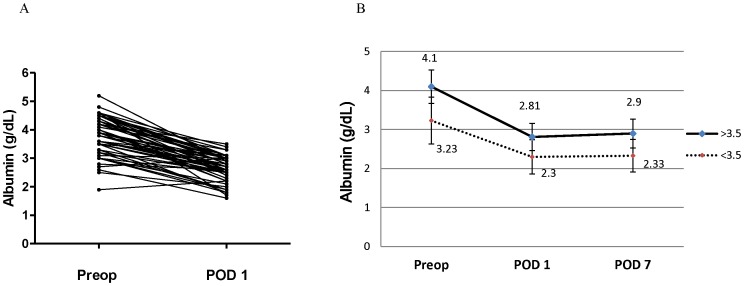
Serial change of albumin levels in the first week postoperatively. (A) POD1 albumin concentrations were significantly lower than preoperative albumin concentrations for all patients (*p*<.0001). (B) The mean albumin concentration declined postoperatively in both the group, but the group with preoperative albumin levels >3.5 g/dL maintained a higher mean postoperative albumin level. The lowest albumin concentrations were uniformly seen on POD1 in the early period postoperatively. (Preop: preoperative; POD1: postoperative day 1; POD7: postoperative day 7).

In this study, POD1 albumin concentrations were significantly lower than preoperative albumin concentrations (*p*<.0001) (Fig. 2A). The mean albumin concentration declined postoperatively in both the group with preoperative albumin levels >3.5 g/dL and the group with levels ≤ 3.5 g/dL, but the group with higher preoperative albumin levels maintained a higher mean postoperative albumin level. The lowest albumin concentrations were uniformly seen on POD1 (Fig. 2B).

### Albumin Loss Associated with Increased Blood Loss

**Figure 3:**
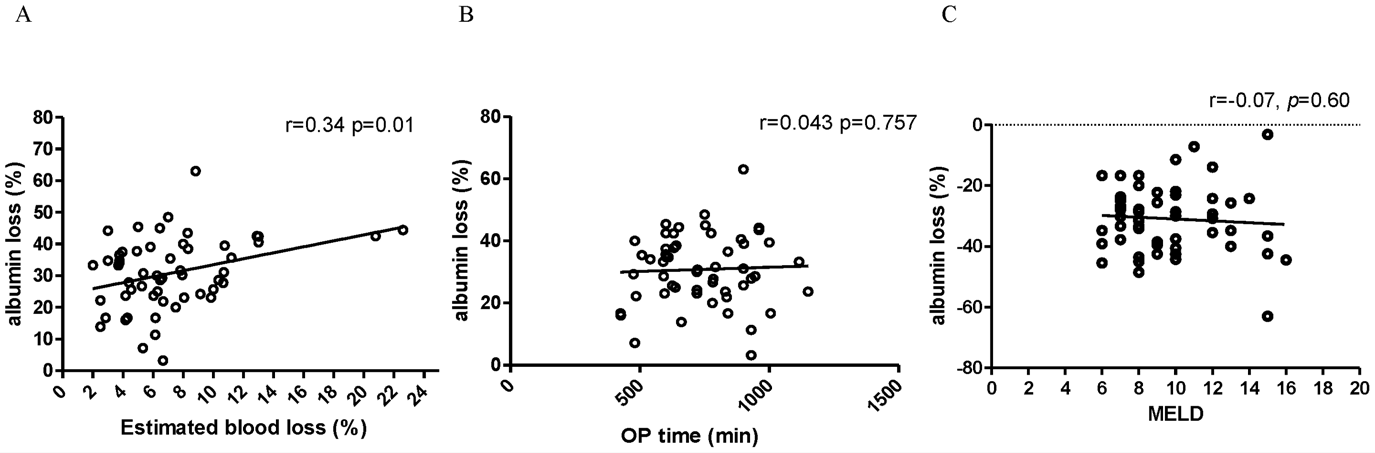
Albumin loss associated with increased blood loss. (A) Significantly positive correlation between the percentages of serum albumin level drops and intraoperative blood loss. (B) & (C) The percentages of serum albumin level drops were not significantly correlated with operative time or MELD scores.

There was a significantly positive correlation between the percentages of which the serum albumin level drops and intraoperative blood loss (*r* = 0.34, *p* = 0.01) (Fig. 3A). However, the percentage of albumin level drops was not significantly correlated with operative time (*r = *0.043, *p = *0.757) or MELD score (*r* = −0.07, *p* = 0.60) (Fig. 3B & C).

### Low Preoperative and POD1 Albumin Associated with Increased Postoperative Morbidity and Mortality

Overall complication rate, combining both medical and surgical complications, was 63.2% (36 patients). Surgical complications occurred in 52.6% (30 patients), including flap loss (partial or total), neck hematoma, postoperative infection, wound dehiscence, and orocutaneous fistula. No difference was seen between the rates of surgical complication in patients with normal preoperative albumin levels of >3.5 g/dL and those with levels ≤ 3.5 g/dL (n = 18 (47.4%) vs. 12 (63.2%), *p* = 0.39). However, the risk of postoperative surgical complication was significantly higher in patients with POD1 albumin ≤ 2.7 g/dL than those with levels >2.7 g/dL (*p* = 0.02; Table 3).

**Table 3:** Postoperative morbidities and mortality.

	Preop^a^ Albumin (g/dL)			POD1^b^ Albumin (g/dL)		
	>3.5	≤ 3.5	*p* value	>2.7	≤ 2.7	*p* value
	(n = 38)	(n = 19)		(n = 30)	(n = 27)	
**Surgical complication**						
Vessel occlusion	3	0	0.54	0	3	0.10
Neck hematoma with exploration	1	7	< 0.01*	1	7	0.02*
Flap loss	3	1	1.00	1	3	0.34
Wound infection, orocutaneous fistula	15	10	0.40	10	15	0.11
Overall surgical complication	18	12	0.40	12	18	0.02*
**Medical complication**						
Cardiovascular (AMI^c^/CAD^d^)	0	1	0.33	0	1	0.47
Lung (pleural effusion, pneumonia, ARDS^e^)	6	11	< 0.01*	3	14	< 0.01*
GI (UGI^f^ bleeding)	1	3	0.10	1	3	0.34
Kidney (acute renal failure)	2	5	0.03*	0	7	< 0.01*
Sepsis	2	6	0.01*	0	8	< 0.01*
Encephalopathy	4	5	0.14	4	5	0.72
Overall medical complication	9	13	< 0.01*	7	15	0.02*
**Mortality**	1	6	< 0.01*	0	7	< 0.01*

a Preop, preoperative;

b POD1, postoperative day 1;

c AMI, acute myocardial infarction;

d CAD, coronary artery disease;

e ARDS, acute respiratory distress syndrome;

f UGI, upper gastrointestinal;

* *P*<0.05.

* *P*<0.05.

38.6% (22 patients) had medical complications, including pulmonary, acute myocardial infarct, gastrointestinal bleeding, encephalopathy, renal failure and sepsis (Table 3). These major medical complications statistically increased in patients with preoperative albumin ≤ 3.5 g/dL over those with albumin >3.5 g/dL (n = 13 (68.4%) vs. 9 (23.7%), *p* = 0.002) as well as in patients with POD1 albumin ≤ 2.7 g/dL over those with albumin >2.7 g/dL (n = 15 (55.6%) vs. 7 (23.3%), *p* = 0.02). The occurrences of pulmonary complications, renal failure, and sepsis increased significantly in patients with preoperative albumin ≤ 3.5 g/dL over those with preoperative albumin >3.5 g/dL (n = 11 (57.9%) vs. 6 (15.8%), *p* = 0.002; n = 5 (26.3%) vs. 2 (5.3%), *p* = 0.035; n = 6 (31.6%) vs. 2 (5.3%), *p* = 0.013, respectively). These complication rates were also significantly higher for patients with POD1 albumin >2.7 g/dL than those with POD1 albumin ≤ 2.7 g/dL. However, the occurrence of acute myocardial infarction, encephalopathy, and gastrointestinal bleeding did not show significant inter-group differences. In this series, seven patients expired postoperatively, giving an in-hospital mortality rate of 12.3%. The mortality rate was significantly higher in patients with preoperative albumin ≤ 3.5 g/dL than those with albumin >3.5 g/dL (n = 6 (31.6%) vs. 1(2.6%), *p* = 0.004; Table 3). Moreover, all in-hospital mortality (100%) occurred in the subgroup of patients with POD1 levels ≤ 2.7 g/dL (none in the subgroup with POD1 levels >2.7 g/dL).

### Preoperative Albumin Concentration as a Predictor of Postoperative Medical and Surgical Morbidity and In-hospital Mortality

The logistic-regression model was used to evaluate the risks of preoperative clinical factors for prediction of postoperative morbidities and mortality. In a mulivariate analysis adjusted for age, sex, hemoglobin levels, and BMI, we found that lower preoperative albumin levels were significantly associated with a higher risk of medical and surgical morbidities and in-hospital mortality (Table 4).

**Table 4:** Multivariate analysis of risks for postoperative morbidity and mortality.

Parameter	Preop serum albumin (g/dL) ≤3.5 vs. >3.5		POD 1 serum albumin (g/dL) ≤2.7 vs. >2.7	
	Adjusted OR (95% CI)	*P* value	Adjusted OR (95% CI)	*P* value
Medical morbidity	4.84 (1.27–18.50)	0.02*	3.56 (1.00–12.67)	0.04*
Surgical morbidity	2.66 (0.71–9.99)	0.15	4.79 (1.30–17.60)	0.02*
Mortality	25.43 (2.02–320.69)	0.01*	N.A.	N.A.

Preop: pre-operative; POD1: post-operative day 1.

Multivariate logistic regression model adjusted with age, sex, preoperative hemoglobin levels, and BMI.

N.A.: Because all in-hospital mortality (100%) occurred in the subgroup of patients with POD1 levels ≤ 2.7 g/dL (none in the subgroup with POD1 levels >2.7 g/dL), the odd ratio becomes infinity and can’t be accurately estimated by logistic regression model.

* *P*<0.05.

### POD1 Albumin Concentration as a Predictor of Postoperative Medical Morbidity and In-hospital Mortality

The logistic-regression model was also used to evaluate the risks of POD1 levels for postoperative morbidity and mortality. Similarly, in a mulivariate analysis adjusted for age, sex, hemoglobin levels, and BMI, we found POD1 albumin levels were significantly associated with higher risk of medical and surgical morbidities (Table 4). Because all in-hospital mortality (100%) occurred in the subgroup of patients with POD1 levels ≤ 2.7 g/dL (none in the subgroup with POD1 levels >2.7 g/dL), the odds ratio becomes infinity and can’t be accurately estimated by logistic regression model. Nonetheless, the patients with POD1 levels are definitely the vulnerable group susceptible to in-hospital mortality.

## Discussion

Albumin is frequently used as an indicator of nutritional status. With its half-life of 20 days, its serum concentration reflects a patient’s nutritional status over a sustained period of time. Hypoalbuminemia has been demonstrated to more reliably reflect protein-energy malnutrition than anthropomorphic markers in many studies [7], [8]. There is convincing evidence that the lower the serum albumin level, the higher the risk for postoperative complications and death [8]–[11].

In patients undergoing total laryngectomy, Schwartz et al. and Gonzalez et al. found that preoperative hypoalbuminemia was independently associated with postoperative wound infection [12], [13]. To our knowledge, this is the first study evaluating the changes of serum albumin levels during peri-operative period, and correlating these changes to surgical outcomes, postoperative morbidity and mortality in head and neck cancer patients with cirrhosis.

The presence of hypoalbuminemia has been thought to be the result of nutritional depletion secondary to the tumor and to be associated with tumor size and site of disease in patients with advanced cancer [14]. In head and neck cancer patients, the perioperative change of nutritional status is associated with a history of heavy alcohol consumption, local tumor effect, poor diet practices, anorexia, and treatment effects [1]. However, the relationship between preoperative serum albumin level and severity of liver cirrhosis has not been established in this specific group. During a 9-year follow-up, the incidence of hypoalbuminemia in head and neck cancer patients with cirrhosis was 33.3%. The preoperative albumin levels were correlated inversely with the MELD scores. Interestingly, no difference in tumor staging was found between the group with normal albumin levels (>3.5 g/dL) and the group with low albumin levels (≤3.5 g/dL). This suggested that the presence of cirrhosis had a dominating influence on the preoperative albumin levels over tumor stage in this specific group of patients (Fig. 1B).

Our data demonstrated a uniform decrease in albumin levels in all patients during the immediate postoperative period, with the lowest levels seen between 6 to 12 postoperative hours (Fig. 2). We believe that the albumin decrease was secondary to following factors: 1) albumin was lost along with blood during surgery; 2) hemodilution from massive intraoperative fluid loading; 3) postoperative albumin extravasation due to increased vascular permeability from a systemic inflammatory response incited by prolonged surgery.

Both groups of patients, those with normal preoperative albumin levels (>3.5 g/dL) and those with low albumin levels (≤3.5 g/dL), developed significant decreases in albumin levels within 1 day from surgery. The percentages of decrease were 27.1±12.9% and 32.7±9.8%, respectively. The difference, however, carried no statistical significance (*p* = 0.1). A positive correlation was observed between the percentages of albumin decrease and intraoperative blood losses in both groups (Fig. 3A). This suggested that the larger the intraoperative blood loss was, the lower the postoperative albumin level would be. This result also revealed that the dilution of plasma albumin through blood loss with massive transfusion, especially when the use of vasoactive pressors was avoided in these free flap cases, were thought to be the main cause of early postoperative hypoalbuminemia rather than the stress-induced weakening of the liver’s ability to synthesize albumin brought on by this major operation. Further research to clarify the underlying mechanism is required to provide a better understanding of the therapeutic implications.

In particular, having a low POD1 albumin level was found to be especially predictive of adverse surgical outcomes, more so than the preoperative hypoalbuminemia. Hypoalbuminemia is associated with poor tissue healing, decreased collagen synthesis in surgical wounds, and impairment of immune responses, such as macrophage activation and granuloma formation [13], [15]–[17]. The compromised systemic immune status then predisposes patients to surgical infection.

Patients with hypoalbuminemia (preoperative levels ≤ 3.5 g/dL or POD1 levels ≤ 2.7 mg/dL) tended to have prolonged ICU stay, which may be a reflection of higher rates of postoperative complications in these patients. A close to fourfold increase in risk of medical complications was seen in these patients as well, with major differences seen in the rates of sepsis, acute renal failure, and pulmonary complications. The most common postoperative medical complication was pleural effusion, occurring in 38.6% of the patients. This is distinctly high when compared to the pleural effusion incidence of 5–10% in patients with only cirrhosis.^18^ Postoperative pleural effusion might develop as a result of transdiaphragmatic movement of ascites from the peritoneal cavity, assisted by the negative intra-thoracic pressure [18]. In our study, 11 of 13 patients with preoperative ascites developed postoperative pleural effusion. This finding demonstrated a strong correlation between preoperative ascites and postoperative pleural effusion, reminding the clinicians the potential pulmonary complications which might be encountered in dealing with the patients with liver cirrhosis and ascites.

Acute renal failure (ARF) is a relatively frequent problem, occurring in approximately 20% of hospitalized patients with cirrhosis [19]. In this series, there were 7 patients suffered from ARF postoperatively with the incidence of 12.3%. Despite significant improvements in intensive care medicine, the development of postoperative acute renal failure in head and neck cancer patients with cirrhosis was an ominous sign, carrying a mortality rate of 100% in our study.

### Conclusion

In our assessment of the prognostic value of serum albumin concentration in head and neck cancer patients with liver cirrhosis, we demonstrated that preoperative serum albumin concentration ≤ 3.5 mg/dL as well as POD1 albumin concentration ≤ 2.7 mg/dL was an independent predictor of postoperative medical complication and mortality. In particular, patients with POD1 albumin concentration ≤ 2.7 mg/dL had a stronger association with increased surgical complications. Therefore, to improve the postoperative medical and surgical outcomes in head and neck patients with liver cirrhosis, physicians should consider the potential role and serial changes of serum albumin level.
